# Front-of-Pack Nutrition Labels: Comparing the Nordic Keyhole and Nutri-Score in a Swedish Context

**DOI:** 10.3390/nu15040873

**Published:** 2023-02-09

**Authors:** Stephanie Pitt, Bettina Julin, Bente Øvrebø, Alicja Wolk

**Affiliations:** 1 Institute of Environmental Medicine, Karolinska Institutet, 17177 Stockholm, Sweden; 2 Department for Sustainable Diets, Swedish Food Agency, 75126 Uppsala, Sweden; 3 Department of Food Safety, Norwegian Institute of Public Health, 0456 Oslo, Norway

**Keywords:** front-of-pack nutrition label, FOPNL, Nutri-Score, Keyhole, Sweden, Europe

## Abstract

The extent to which different front-of-pack nutrition labels (FOPNLs) agree or contradict each other has been insufficiently investigated. Considering the 2020 proposal from the European Commission to create a harmonized FOPNL, the aim of this study was to assess agreements and disagreements between two FOPNL schemes—the Keyhole and the Nutri-Score—in a Swedish context. The current Keyhole criteria and the updated Nutri-Score 2022 algorithm were applied to 984 food items and their nutrient compositions, obtained from the food database of the Swedish Food Agency. Agreements (Keyhole-eligible and Nutri-Score A or B; or not Keyhole-eligible and Nutri-Score C, D, or E) and disagreements (Keyhole-eligible and Nutri-Score C, D, or E, or not Keyhole-eligible and Nutri-Score A or B) were calculated as percentages for all items and by food group. An agreement was found for 81% of included items. The lowest level of agreement was found for the groups of flour, grains, and rice (62% agreement) and for plant-based meat and fish analogues (33% agreement). There is generally a good level of agreement between the Keyhole and the Nutri-Score for food items on the Swedish market. Large disagreements found for plant-based meat and fish analogues, and products based on cereals/grains, highlight important considerations for the development of a harmonized FOPNL within Europe.

## 1. Introduction

Western diets—predominant across parts of Europe and North America—are typically characterized by a low intake of fruit, vegetables, and whole grains, and a high intake of processed foods, refined grains, salt, sugar, and saturated fats. The increased consumption of such foods in the diet, combined with low levels of physical activity, is associated with the increasing prevalence of overweight and obesity [1] which is an established risk factor for the development of non-communicable diseases [2]. Approaches to generating both population- and individual-level transitions toward healthier diets are therefore necessary.

Providing nutritional information on food packaging is an example of a macro-level measure that aims to improve population-level diet. Front-of-pack nutrition labels (FOPNLs) have been identified by the World Health Organization (WHO) as an important policy tool to guide consumers towards making healthier food choices [3], whilst also encouraging manufacturer-led product reformulation. The overall goal of an FOPNL is to provide at-a-glance nutritional information at the point of purchase, thus enabling consumers with different health literacy [4] to better distinguish between food products with high or low nutritional value.

Pre-packed foods sold within the European Union (EU) are required to display a nutrient declaration, usually found on the back of the pack [5]. However, as of today, there is no harmonized approach to front-of-pack nutrition labelling within the EU. As such, considerable variations in both labelling schemes and the associated terminology exist [6]. Generally, FOPNLs fall into one of two categories—”nutrient-specific” (e.g., reference intake labels) or “summary indicators”, which can be further divided into:

i. Positive endorsement labels (e.g., the Nordic Keyhole);
ii. Warning labels (e.g., the Chilean warning label);
iii. Graded indicator labels (e.g., the Nutri-Score).

Despite there being multiple forms, there is limited evidence on the effects of FOPNLs on purchasing behaviours and improvements in dietary intake and health outcomes [6], particularly for some groups, such as people suffering from eating disorders [7]. However, within EU countries, FOPNLs are generally appreciated by consumers, and, in comparison to no label, most have been found to have a positive influence on the ability of consumers to identify the healthier food choice [6,8]. Therefore, as part of the Farm to Fork strategy adopted by the European Commission in 2020, the introduction of an EU-wide harmonized and mandatory FOPNL has been proposed [9]. Further details on the proposal were expected by the end of 2022, although there has been no confirmation as of January 2023.

### 1.1. Nordic Keyhole

**Figure 1:**
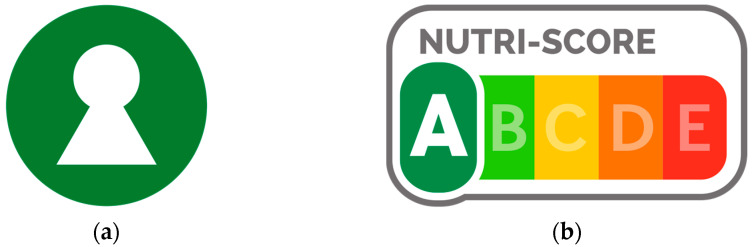
Two front-of-pack nutrition labels (FOPNLs): (**a**) the Keyhole, a positive endorsement FOPNL, owned by the Swedish Food Agency (*Livsmedelsverket*). The Keyhole—which has been implemented in Sweden since 1989, and more recently in Denmark, Iceland, Lithuania, Norway, and North Macedonia—indicates a healthier food option in comparison to foods within the same category; (**b**) the Nutri-Score, a graded indicator FOPNL, owned by Santé publique France. The Nutri-Score, originating in France in 2017 and more recently implemented in Belgium, Germany, Luxembourg, the Netherlands, Spain, and Switzerland, provides an across-the-board indication of the healthfulness of a food item, with scores ranging from A (high nutritional value) to E (low nutritional value).

The proposal is supported by the Nordic countries [10], in four of which the Nordic Keyhole (hereafter, Keyhole) is the adopted FOPNL (excluding Finland). Criteria for determining which food items are eligible to display the Keyhole are based on the Nordic Nutritional Recommendations, which constitutes the scientific basis for national nutrient recommendations and food-based dietary guidelines in the Nordic countries [11,12]. The Keyhole is a positive endorsement logo (Figure 1), meaning that it indicates when a food item or product is a healthier option in comparison to other products in the same category (e.g., less salt, lower in sugars, contains more fibre and whole grains, or contains healthier or less fat). One study found that by replacing some non-eligible food items with equivalent food items eligible to be labelled with the Keyhole, an improvement in meeting nutritional recommendations was achieved [13]. Furthermore, consumer awareness in Sweden is estimated to be high, with 97% of 18–80-year-olds reporting being familiar with the Keyhole symbol [14]. However, it is also reported that few consumers have a deeper insight or understanding of what the Keyhole symbol represents in practice [15].

The Keyhole was introduced as an FOPNL in Sweden in 1989, and it has been adopted by five other countries as of January 2023: Denmark, Iceland, Lithuania, Norway, and North Macedonia [16]. The Swedish Food Agency (*Livsmedelsverket* in Swedish) is a government agency that is the brand owner of the Keyhole, although the use of the Keyhole is voluntary and at the discretion of the food manufacturer. However, products with a low nutritional value (e.g., salted or sweet snacks and pastries), or those containing artificial sweeteners, plant sterols, or more than 2% industrially produced trans fatty acids cannot be labelled with the Keyhole. No registration is necessary for the use of the Keyhole and in Sweden, eligibility must be determined by the manufacturer with support offered by the Swedish Food Agency. Use of the Keyhole is then controlled by the responsible authority, usually at the municipality level.

### 1.2. Nutri-Score

More recently, several European countries have adopted the Nutri-Score FOPNL (Figure 1): a coloured five-letter grading system used to demonstrate the overall nutritional value of any given product, excluding unprocessed products comprising of a single ingredient (e.g., a piece of fruit or cut of raw meat) [17,18]. Originally adapted from the nutrient profiling system developed by the British Food Standards Agency, it was introduced in France in 2017. Since 2017, the Nutri-Score has been adopted by Belgium, Germany, Luxembourg, the Netherlands, Spain, and Switzerland. Similarly to the Keyhole, the Nutri-Score is a voluntary FOPNL. In contrast to the Keyhole, the use of the Nutri-Score label requires manufacturers to register their brand, with all products under the brand also required to use the Nutri-Score label. At present, the available literature suggests that the Nutri-Score (or a similar five-colour nutrition label) may be the most effective FOPNL for improving consumer behaviour, by guiding consumers toward healthier food choices [6], improving the identification of healthier foods [19,20,21] and, as it is a highly interpretative label, requiring the lowest cognitive workload [22]. Furthermore, in comparison to less interpretative labels, one study found the Nutri-Score could improve the understanding of the nutritional value of foods across income levels, thus indicating it is an equitable label [23]. However, the scientific committee of the Nutri-Score identified potential improvements to the algorithm behind the Nutri-Score, enabling it to better align with food-based dietary guidelines. An update of the Nutri-Score algorithm was thus published in 2022 [24], although as of January 2023, it remains under review with the original algorithm [17] currently implemented.

### 1.3. Research Gap and Aim

The Keyhole and the Nutri-Score represent two FOPNLs in use throughout Europe at present. However, the extent to which the labels agree or contradict each other (in terms of how food products are labelled by either scheme) has been insufficiently investigated, particularly for the Nutri-Score 2022 algorithm. Therefore, the aim of this study is to assess the extent of agreement and disagreement between the Keyhole and the Nutri-Score when applied to food items available in Sweden. Assessing the agreements and disagreements between the two schemes could be useful for a better understanding of how an EU-wide harmonized label could be achieved, what challenges may exist, and how this may impact the current labelling scheme in any given context.

## 2. Materials and Methods

### 2.1. Data on Food Items

The Swedish Food Agency food database (version 2022-05-24) was used as a source of food items and their nutritional composition (e.g., saturated fat, protein, and salt) per 100 g [25]. The food database consists of over 2000 food items—most of which are generic, but some are brand-specific—and aims to represent food items available on the Swedish market. Both whole foods (e.g., a piece of fruit) and composite foods (e.g., manufactured products), as well as cooked dishes, are included in the food database. Some food items in the database could not be included in this study. All home-cooked items (e.g., pasta boiled with salt) were excluded, as such items are not representative of what is found on supermarket shelves. Beverages and other drinks (including milk and plant-based alternatives) were also excluded, as the Nutri-Score 2022 algorithm had not been updated to include such items at the time of conducting this study [24]. Items for children under 36 months (e.g., baby food) were excluded as these items are not eligible for either the Keyhole or Nutri-Score FOPNL.

The remaining items were then grouped into one of 11 main food groups, as specified in the current Keyhole eligibility criteria (*Livsmedelsverkets föreskrifter om användning av symbolen Nyckelhålet* (LIVSFS) 2005:9) [11,12]. Since milk and yoghurt-based drinks were excluded, the group containing milk (group 4) was re-named in this study to avoid confusion. Any items that were not included in one of the 11 groups specified by the Keyhole eligibility criteria (e.g., jam and marmalade, pastries, biscuits, and cakes) were placed in an additional group (group 12, “other”), so as to include a wider range of items in the assessment. The 12 food groups were thus as follows:

1 Vegetables, fruits, berries, and nuts;
2 Flour, grains, and rice;
3 Porridge, bread, and pasta;
4 Milk, fermented products, and related plant-based products—hereafter referred to as fermented products and related plant-based products, since milk- and yoghurt-based drinks and related plant-based milk products were excluded from the assessment;
5 Cheese and related plant-based products;
6 Fats, oils, and spreads;
7 Fish, shellfish, and derivative products;
8 Meat and meat products;
9 Plant-based products—with the same range of use as meat or fish in group 7 or 8, hereafter referred to as plant-based meat and fish analogues;
10 Ready meals;
11 Dressings and sauces;
12 Other—this group is not included in the current Keyhole eligibility criteria.

The aim was to include approximately 100 items for each of the groups. However, for some groups, data were available for fewer than 100 items. Overall, 984 items were included in the assessment, covering most of the non-excluded items available from the food database. A second database (referred to as the ingredient database) containing ingredients (i.e., recipes) and their proportions (in percentage) for each item in the food database was provided by the Swedish Food Agency and linked via a unique number to each of the included items. The Keyhole eligibility for each item was subsequently determined, using the current criteria (LIVSFS 2005:9) [11,12], along with the corresponding Nutri-Score, using the updated Nutri-Score 2022 algorithm [24]. Each is described in turn, below.

### 2.2. Application of the Keyhole Eligibility Criteria

Prior to applying the Keyhole eligibility criteria, some minor adjustments or calculations were required. For vegetable items, a criterion is given for added fat; however, the amount of added fat was not available from the food database. Therefore, this criterion was only applied to the vegetable items that had oil/fat listed as an ingredient, as this indicated that fat was added to the item. In all other cases, the items were either 100% vegetable (i.e., had no added fat), or consisted of water (e.g., for canned vegetables) or salt. For items in the groups of flour, grains, and rice; porridge, bread, and pasta; plant-based meat and fish analogues; and ready meals, the amount of whole grain was calculated as a percentage of the dry matter content or as a percentage of total cereal content, whichever was specified by the criteria for the group. For fish items, the proportion of non-fish fat was determined by subtracting polyunsaturated fats from total fat. Where necessary, the proportion of fish and meat in food items was also determined using the ingredient database. The total fruit, vegetable (excluding potato), legume (excluding peanut), and grain (when required by the Keyhole criteria) percentage for all items were determined by summing up the percentages of relevant ingredients.

Items eligible for the Keyhole were required to fit into one of the 11 food groups, as specified by the current Keyhole eligibility criteria, listed above. These items were then further subdivided into one of 32 categories (Table A1 in Appendix A). Products were eligible to be marked with the Keyhole provided the criteria were met (e.g., amount of fibre, whole grain, fat, saturated fat, sugar, and salt). The criteria vary between each of the 32 food categories. The criteria for each category are provided in the Supplementary Material (File S1), with the official criteria reported in detail elsewhere [11,12].

### 2.3. Application of the Nutri-Score 2022 Algorithm

The Nutri-Score 2022 algorithm was applied to all items. More detail on the 2022 algorithm can be found in the Supplementary Material (File S2), with the official report found elsewhere [24]. A brief overview is provided herein. Items were first placed into one of two groups: solid food or fats, oils, nuts, and seeds (including cream products). Within the solid food group, cheese products and red meats were identified, as specific considerations are given to these items in the final calculation. The proportion of fruit, vegetable (excluding potato), and legume (FVL) for each item was determined using the ingredient database. For the fats, oils, nuts, and seeds category, oils derived from vegetables or fruits (e.g., olive or avocado oil) were also identified. In practice, the Nutri-Score is not applied to unpackaged items without a nutrient declaration (e.g., an apple, or a cut of raw meat). However, for the purposes of comparison in this study, the Nutri-Score 2022 algorithm was applied to such items.

In accordance with the Nutri-Score 2022 algorithm, points were given for “favourable” and “unfavourable” elements [24] for each item. The elements comprising favourable and unfavourable elements are shown in Table 1, separately for the two groups (solid foods and fats, oils, nuts, and seeds) with more information on point allocation for each element provided in the Supplementary Material (File S2). Note that for red meat items, the maximum number of points that the protein element can receive is 2. Together, the points for the favourable elements form the *C component,* and the points for the unfavourable elements form the *A component*. The Nutri-Score for each item was then calculated by applying one of the formulas listed below.

For items in the solid food group

If the *A Component* was ≥11 points, then:

*Formula i*. $\mathrm{Nutri}-\mathrm{Score}=A\;Component-\left(Points\;FVL+Points\;Fibre\right)$ (not including points for protein)

If the *A Component* was <11 points or if calculating for cheese, then:

*Formula ii*. Nutri-Score=A Component−C Component

For items in the fats, oils, nuts, and seeds group

If the *A Component* was ≥7 points, then:

*Formula iii*. $\mathrm{Nutri}-\mathrm{Score}=A\;Component-\left(Points\;FVL+Points\;Fibre\right)$ (not including points for protein)

If the *A Component* was <7 points, then:

*Formula iv*. Nutri-Score=A Component−C Component

The final number of points determined the Nutri-Score letter and colour, ranging from the highest nutritional quality labelled with the letter “A” (dark green); to the lowest nutritional quality labelled with the letter “E” (dark orange) (Table 2).

### 2.4. Assessment of Agreement and Disagreement

The number of items (*n*) and percentage of items (%) with Keyhole eligibility (yes/no) and Nutri-Score (A or B/C, D, or E) were determined overall, and within each food group. To equate Keyhole eligibility and the Nutri-Score, an agreement was determined as being Keyhole-eligible and having a Nutri-Score of A or B, or not Keyhole-eligible and having a Nutri-Score of C, D, or E (Table 2). A disagreement between the two was determined as being Keyhole-eligible and having a Nutri-Score of C, D, or E, or not being Keyhole-eligible and having a Nutri-Score of A or B. Descriptive statistics (%) were used to assess the extent of agreement and disagreement overall, and within each group. The percentage of agreement within each group was determined, as well as the percentage of disagreement, separated by the reason for disagreement (i.e., not Keyhole-eligible, but with a Nutri-Score of A or B, or Keyhole-eligible, but with a Nutri-Score of C, D, or E). Stata 16.1 was used for all statistical calculations. Five items in which a disagreement was found were selected across the food groups to further assess how the application of the Keyhole criteria and updated Nutri-Score 2022 algorithm may have led to the observed disagreement. Both the requirements for Keyhole eligibility and whether the specific criterion was met are reported, as well as the Nutri-Score points for each element. Further information on all food items assessed and their corresponding Keyhole eligibility and Nutri-Score are presented in the Supplementary Material (File S3). For each item, nutrient quantities are presented that are relevant to determining Keyhole eligibility and/or the Nutri-Score. In addition, the individual Nutri-Score points given for both the favourable and unfavourable elements are shown.

## 3. Results

Across the 984 items that the current Keyhole criteria and Nutri-Score 2022 algorithm were applied to, 36% were found to be Keyhole-eligible. For the Nutri-Score, 48% of items received a score of A or B (Table 3). The group of vegetables, fruits, berries, and nuts had the highest percentage of items eligible for the Keyhole (76%), as well as the highest percentage of items with a Nutri-Score of A or B (90%). For three groups—fats, oils, and spreads; fish, shellfish, and derived products; and meat and meat products—a higher percentage of items were found to be Keyhole-eligible, compared to items that received a Nutri-Score of A or B. For one group—cheese and related plant-based products—the number of items eligible for the Keyhole was the same as the number of items with a Nutri-Score of A or B. For the remaining eight groups, a higher percentage of items were found to have a Nutri-Score of A or B, compared to the proportion of items that were Keyhole-eligible.

An agreement between the two FOPNLs was found for 81% of items (*n* = 799) whereas a disagreement was found for 19% (*n* = 185) (Table 4). Of the 799 items for which an agreement was found, 40% was due to being Keyhole-eligible and having a Nutri-Score of A or B. Therefore, 60% of items for which an agreement was found were not Keyhole-eligible and had a Nutri-Score of C, D, or E. Of the 185 items for which a disagreement was found, 83% was due to being not Keyhole-eligible, but having a Nutri-Score of A or B. Thus, 17% of items for which a disagreement was found were Keyhole-eligible, but had a Nutri-Score of C, D, or E.

Within 8 of the 12 food groups, an agreement between the Keyhole and Nutri-Score was found for at least 80% of all items (Table 4). The greatest agreement between the two schemes was found for the following groups (% agreement): dressings and sauces (97%); meat and meat products (90%); fish, shellfish, and derived products (88%); cheese and related plant-based products (88%); vegetables, fruits, berries, and nuts (85%); ready meals (81%); and fats, oils and spreads (80%). In addition, a high level of agreement (97%) was found within the “other” group, which includes items such as salted and sweet snacks and pastries. The lowest level of agreement between the Keyhole and the Nutri-Score was found for the following groups (% agreement): porridge, bread, and pasta (70%); fermented products and related plant-based products (63%); flour, grains, and rice (62%); plant-based meat and fish analogues (33%).

Details of why there are differences between some items are shown for the examples provided in Table A2 (see Appendix B). For instance, for the group of plant-based meat and fish analogues—for which there was least agreement—the item *soy protein kebab* was not eligible for the Keyhole due to its high salt content. However, in the application of the Nutri-Score, the points resulting from the high salt content were balanced out by highly favourable elements of protein and fibre, and thus the item received a Nutri-Score of A. Additionally, in the group of porridge, bread, and pasta, the item *wholegrain bread, rye unsweetened* was not eligible for the Keyhole due to the proportion of whole grain based on dry matter content being less than 30%, but the item received a Nutri-Score of B. For the group of cheese and related plant-based products, a disagreement was also found for *hard cheese*, *17% fat*. In this case, the item was eligible for the Keyhole, despite the highly unfavourable elements of saturated fat and salt, which were not outweighed by a favourable protein content, and resulted in a Nutri-Score of D.

## 4. Discussion

The aim of this study was to identify the extent to which the Keyhole and the Nutri-Score were in agreement or disagreement when applied to food items in the Swedish context. Of the 984 items included in the assessment, 36% were found to be eligible for the Keyhole, whilst 48% were able to receive a Nutri-Score of A or B. Of the 984 items, an agreement between the Keyhole and Nutri-Score (i.e., Keyhole-eligible and a Nutri-Score of A or B, or not Keyhole-eligible and a Nutri-Score of C, D, or E) was found for 81% of items. Of the items for which a disagreement was found, the Keyhole appeared to be more restrictive, with 83% of disagreements due to not being Keyhole-eligible, but having a Nutri-Score of A or B. The least agreement was found for the groups of fermented products and related plant-based products; flour, grains, and rice; and plant-based meat and fish analogues.

### 4.1. Interpretation of Results

Overall, there appears to be a good agreement between the current Keyhole criteria and the updated Nutri-Score 2022 algorithm, when applied to food items on the Swedish market. This is particularly true for food groups at either end of the scale from low to high nutritional value. For instance, a high level of agreement was found for fruits and vegetables (generally high in nutritional value) and for pre-packaged dressings and sauces (generally low in nutritional value). Based on these findings, for most items on the Swedish market, a hypothetical introduction of the Nutri-Score is unlikely to generate profound changes to the indicated healthfulness of a large number of products.

In terms of which items could be labelled as a healthier option, the findings from the presented study indicate that the Keyhole appears to be more restrictive compared to the Nutri-Score. One explanation for this could be the category-specific criteria for determining Keyhole eligibility, as opposed to an across-the-board application (as utilized in the Nutri-Score algorithm). Therefore, for some items on the Swedish market, a hypothetical introduction of the Nutri-Score 2022 algorithm would result in some currently not Keyhole-eligible items receiving a Nutri-Score of A or B. This highlights a difference in the aim of each label. While both provide an at-a-glance indication of nutritional quality, the primary aim of the Keyhole is to enable consumers to identify items with a higher nutritional quality in comparison to other products of the same category (which are usually placed on the same shelf). Conversely, the Nutri-Score aims to provide an overall indication of the nutritional quality of an item. In practice, though, the Nutri-Score is also applied within categories (e.g., when choosing among cereals, the label guides consumers towards choosing the item with a better Nutri-Score).

In terms of the application of both FOPNLs, the Keyhole is applicable only to items that can fit into the pre-determined 11 food groups, whereas the Nutri-Score is (in practice) applied only to pre-packaged items with a nutrient declaration, thus excluding unprocessed fruits, vegetables, meat, and fish. Despite this difference in application, a general agreement in the Keyhole eligibility and Nutri-Score across these food items was found, as shown by the high percentage of agreement in the groups containing fruit and vegetables, meat, and fish.

However, a lack of agreement between the schemes in certain groups indicates two differences. First, the perceived healthfulness of plant-based meat and fish analogues currently differs, since this was the group with the least agreement found. Plant-based meat and fish analogues can be challenging to generalize from a health perspective since their nutritional compositions can vary substantially between products [26] and certain properties, such as reduced bioavailability of iron, have only recently been explored [27,28]. Nonetheless, increased consumption of plant-based proteins at the expense of animal-based proteins has been suggested to improve longevity [29] and reduce climate impact [30]. Thus, improved coherence with regard to the labelling of this food group is important, as well as the ability of consumers to identify healthier plant-based meat and fish analogues, particularly considering their increasing sales [31]. This is also relevant from a sustainability perspective since plant-based foods generally carry a lower environmental impact in comparison to animal products [32]. Second, the use of whole grain or fibre as an indicator of healthfulness differs, since less agreement was found for groups in which Keyhole eligibility is in part determined by the whole grain content (e.g., flour, grains, and rice, as well as porridge, bread, and pasta). This finding was expected, since the Keyhole has certain requirements for whole grain and fibre in some instances, whereas the Nutri-Score does not have a whole grain requirement but rather uses fibre content as a proxy. This is further exemplified in Table A2 (see Appendix B), for the wholegrain bread item in which a disagreement between the two FOPNLs is found based only on the whole grain requirement (or lack thereof). Since the consumption of whole grains is recommended in many food-based dietary guidelines in Europe [33], including whole grain composition in an assessment of the healthfulness of a food item appears important. Given that the definition of whole grain can differ between the EU and its constituent countries [34], this may present a potential challenge to the development of a harmonized label.

### 4.2. Strengths and Limitations

Several strengths of this study have been identified. First, the updated version of the Nutri-Score algorithm, published in 2022, was applied, and thus the findings from this study can provide both relevant and timely points for discussion. Second, an exact assessment (rather than an estimation) of applying both FOPNLs was carried out, including a large number of items, thus maximizing the accuracy of the findings. Third, the use of the food database from the Swedish Food Agency as a source of food items, their composition, and nutritional content, ensures the reproducibility of this study upon data or criteria updates, or to answer future research questions. Fourth, the findings are generalizable to food items not included in the study but belonging to one of the food groups explored. However, although the vast majority of items assessed are common across supermarkets in Europe, some items are local to Sweden, which should be taken into consideration when generalizing the results outside of Sweden.

Despite the strengths of this study, the following limitations should be acknowledged. First, for some food groups only a small number of items were included. For instance, some plant-based meat and fish analogues are relatively new to the market, and thus fewer items than are currently available were included in the food database. This is unlikely to significantly impact the overall findings but does reduce the reliability of the findings for smaller groups. Second, some items in the database are outdated (e.g., analysed in 2012), and other items listed are an average of several similar products. Therefore, the items included in this study may not exactly match the nutritional composition of some items currently on the market. However, the impact on the findings and conclusions drawn is limited, since both the Keyhole and the Nutri-Score were applied to the same items. Third, a limitation may exist in the attempt to equate the two FOPNLs as the interpretation of Keyhole eligibility may not perfectly match with a Nutri-Score of A or B. For the purposes of this study, this was necessary to enable an appropriate comparison.

### 4.3. Similar Studies in the Literature

There is currently limited published literature assessing the extent of agreement and disagreement between applying the Keyhole and the Nutri-Score 2022 algorithm to food items. A Danish report directly compared some aspects of the Keyhole and the Nutri-Score algorithm and provides a useful overview of practical differences [35]. However, this study did not assess the application of either scheme to food items on the market. In addition, the authors did not utilize the Nutri-Score 2022 algorithm. A 2022 study by Konings et al. [36] compared how well two FOPNLs—the Choices five-level criteria, and the Nutri-Score—aligned to current Dutch food-based dietary guidelines. The authors found that the Choices five-level criteria aligned more closely with the food-based dietary guidelines, noting that many discrepancies were found between the Nutri-Score and the guidelines. However, since the updated Nutri-Score 2022 algorithm was not applied, it is challenging to draw comparisons. Söderlund et al. [37] compared two FOPNLs—the Australasian Health Star Rating and the Chilean warning labels—when applied to 13,000+ food items on the market in New Zealand. Whilst the compared FOPNLs differ from those compared in the presented study, the authors found comparable results, with a good level of agreement found in general between the two labelling schemes, but higher levels of disagreement for specific food categories, including cereals and cereal products. The similarity between the studies on this matter supports the interpretation that improved coherence between different FOPNLs may be necessary.

### 4.4. Further Implications

The presented study is theoretical, only considering if an item would be eligible for the Keyhole and which Nutri-Score it could receive. Using these findings as a starting point, further investigation into the uptake of both FOPNLs in different countries, as well as further investigation into the impact on consumer behaviour, could provide an indication of the current situation in different contexts. In addition, broader questions, such as the implication of harmonized FOPNLs on imports/exports are also brought to light. For instance, it is likely that, at present, some Keyhole-eligible food items which are imported to Sweden from within the EU may not display the Keyhole FOPNL, thus potentially limiting the effect of the label. This highlights one benefit of the development of an EU-wide harmonized label.

In the process of conducting this study, some potential points for discussion on how to achieve a successful EU-wide FOPNL have been generated. Alongside promoting healthier diets at the consumer level, FOPNLs can also encourage manufacturer-led product reformulation. For instance, with respect to the Keyhole criteria, concrete cut-off values are given for each element within a category, thus providing specific reasons for a product to be eligible or not. As such, for manufacturers, the Keyhole criteria can serve as a benchmark by providing clear areas for improvement. By contrast, Nutri-Score points are awarded the same way within a group (with special considerations given to cheese and red meat). Therefore, when applying the Nutri-Score, it is more challenging to determine a specific element that results in the final score. For some items, such as the *soy protein kebab* (see Table A2 in Appendix B), a relatively unfavourable salt content (which is a factor resulting in the item not being eligible for the Keyhole) is “offset” by favourable protein and fibre, resulting in a Nutri-Score of A. Consequently, there may be little motivation for manufacturers to reduce the high salt content. This example illustrates a possible benefit of a more granular approach to labelling criteria, as different components are necessary for different micro- and macro-nutrients, and hence may be more or less important depending on the food category. However, the motivation for product reformulation may also vary depending on the food item. For instance, for snack items (e.g., biscuits) there are no criteria to serve as a benchmark, since these items are not eligible for the Keyhole. By contrast, the manufacturer of a food item with a Nutri-Score of D or E could be encouraged to reformulate several aspects of the product to achieve a higher Nutri-Score grading, since points in the Nutri-Score algorithm are given across the board. An improved understanding of the effects of different types of FOPNLs on both manufacturer and consumer behaviour is thus necessary. Overall, this highlights that the discussion on FOPNLs within Europe is not a matter of public health alone, but it involves a geopolitical debate and is also closely coupled with the operation of industry.

## 5. Conclusions

This study aimed to compare the application of two FOPNLs—the Keyhole and the Nutri-Score—to determine to what extent the two schemes agreed or disagreed with respect to the scores given for the nutritional quality of food items available on the Swedish market. The results indicate a generally good level of agreement between the application of the two FOPNLs in most food groups, particularly with respect to items that are known to be high or low in nutritional value. However, disagreements exist within some food groups, particularly plant-based meat and fish analogues, and products based on cereals/grains, in which the discrepancies between whole grain and fibre requirements of the two FOPNLs can be observed. Areas of agreement, and particularly disagreement, are important for discussion when considering a harmonized FOPNL across Europe.

## Figures and Tables

**Table 1:** Distinction between “favourable” and “unfavourable” elements within both food groups (solid foods and fats, oils, nuts, and seeds), as specified by the Nutri-Score 2022 algorithm [24].

For Solid Foods		For Fats, Oils, Nuts, and Seeds	
Favourable Elements	Unfavourable Elements	Favourable Elements	Unfavourable Elements
- FVL proportion (%) - Protein (g/100 g) - Fibre (g/100 g)	- Energy (kg/100 g) - Sugar (g/100 g) - Saturated fat (g/100 g) - Salt (g/100 g)	- FVL proportion (%) - Protein (g/100 g) - Fibre (g/100 g)	- Energy from saturated fats (kg/100 g) - Sugar (g/100 g) - Saturated fat as a percentage of fat (%) - Salt (g/100 g)
Together form the *C component*	Together form the *A component*	Together form the *C component*	Together form the *A component*

FVL: Fruit, vegetable, and legume. For both food groups, points are given for three favourable elements, which together form the *C component*. Points are also given for four unfavourable elements, which together form the *A component*.

**Table 2:** Total Nutri-Score points and corresponding letter and colour for the two food groups in the Nutri-Score 2022 algorithm (solid foods and fats, oils, nuts, and seeds).

Nutri-Score Points for Solid Foods	Nutri-Score Points for Fats, Oils, Nuts, and Seeds	Nutri-Score Letter and Colour		Keyhole Eligibility *For Agreement*
Min. to 0	Min. to −6	A		Yes
1 to 2	−5 to 2	B		Yes
3 to 10	3 to 10	C		No
11 to 18	11 to 18	D		No
19 to Max.	19 to Max.	E		No

The Nutri-Score letter ranges from A (high nutritional value) to E (low nutritional value). An agreement between the Keyhole and Nutri-Score is considered to be Keyhole eligible and Nutri-Score A or B, or not Keyhole eligible and Nutri-Score C, D or E.

**Table 3:** The number of food items included in the assessment and the corresponding Keyhole eligibility and Nutri-Score, separated by group according to the Keyhole criteria (LIVSFS 2005:9).

Group Number and Name		Number of Items *n*	Keyhole Eligibility *n* (%)		Nutri-Score *n* (%)	
			Yes	No	A or B	C, D, or E
1	Vegetables, fruits, berries, and nuts	221	167 (76)	54 (24)	198 (90)	23 (10)
2	Flour, grains, and rice	74	22 (30)	52 (70)	48 (65)	26 (35)
3	Porridge, bread, and pasta	90	15 (17)	75 (83)	42 (47)	48 (53)
4	Fermented products and related plant-based products *	49	5 (10)	44 (90)	23 (47)	26 (53)
5	Cheese and related plant-based products	48	7 (15)	41 (85)	7 (15)	41 (85)
6	Fats, oils, and spreads	46	18 (39)	28 (61)	9 (20)	37 (80)
7	Fish, shellfish, and derived products	68	46 (68)	22 (32)	38 (56)	30 (44)
8	Meat and meat products	121	61 (50)	60 (50)	53 (44)	68 (56)
9	Plant-based meat and fish Analogues **	30	0 (0)	30 (100)	20 (67)	10 (33)
10	Ready meals	104	9 (9)	95 (91)	29 (28)	75 (72)
11	Dressings and sauces	37	1 (3)	36 (97)	2 (5)	35 (95)
12	Other *** (e.g., salted and sweet snacks)	96	0 (0)	96 (100)	3 (3)	93 (97)
	All food items	984	351 (36)	633 (64)	472 (48)	512 (52)

The number of items (*n*) and percentage of items (%) eligible and not eligible for the Keyhole is shown for all items and within groups. The number of items (*n*) and percentage of items (%) receiving a Nutri-Score of A or B and C, D, or E is shown for all items and within groups. * Milk- and yoghurt-based drinks and corresponding plant-based alternatives were excluded from the group. ** With the same range of use as meat or fish in group 7 or 8. *** This group is not included in the Keyhole eligibility criteria.

**Table 4:** The percent (%) of agreement and disagreement for included food items, separated by group according to the current Keyhole criteria (LIVSFS 2005:9).

Group Number and Name		Agreement ^†^ (%)	Disagreement (%)	
			1 ^††^	2 ^††^
1	Vegetables, fruits, berries, and nuts	85	14	0
2	Flour, grains, and rice	62	36	1
3	Porridge, bread, and pasta	70	30	0
4	Fermented products and related plant-based products *	63	37	0
5	Cheese and related plant-based products	88	6	6
6	Fats, oils, and spreads	80	0	20
7	Fish, shellfish, and derived products	88	0	12
8	Meat and meat products	90	2	8
9	Plant-based meat and fish analogues **	33	67	0
10	Ready meals	81	19	0
11	Dressings and sauces	97	3	0
12	Other *** (e.g., salted and sweet snacks)	97	3	0
All food items		81	16	3

The percentage of items for which an agreement or disagreement was found (separated by reason for disagreement) is shown for all items, and within groups. Due to rounding, some row totals do not add up to 100%. **^†^** An agreement was considered as Keyhole-eligible and a Nutri-Score of A or B, or not Keyhole-eligible and a Nutri-Score of C, D, or E. **^††^** A disagreement was considered as either: 1: not Keyhole-eligible, but a Nutri-Score of A or B; or 2: Keyhole-eligible, but a Nutri-Score of C, D, or E. * Milk- and yoghurt-based drinks and corresponding plant-based alternatives were excluded from the group. ** With the same range of use as meat or fish in group 7 or 8. *** This group is not included in the Keyhole eligibility criteria.

## Data Availability

Publicly available datasets were used in this study. Part of this data can be found here: Food database, Swedish Food Agency: https://www7.slv.se/SokNaringsinnehall/ (accessed on 12 December 2022) or upon request from the Swedish Food Agency. The data presented as results in this study are available in the Supplementary Material (File S3).

## Supplementary Materials

The following supporting information can be downloaded at: https://www.mdpi.com/article/10.3390/nu15040873/s1, File S1: Keyhole Eligibility Criteria; File S2: Nutri-Score 2022 Algorithm; File S3: Food Items in Assessment.

## Appendix A. Keyhole Criteria Groups and Sub-Groups

**Table A1:** Main food groups and categories (i.e., sub-groups) included in the current Keyhole eligibility criteria (LIVSFS 2005:9).

#	Main Group	#	Categories (Sub-Groups)
1	Vegetables, fruit, berries, and nuts	1	Vegetables (including potatoes and legumes, excluding peanuts) and unprocessed spices
		2	Unprocessed fruit and berries
		3	Unprocessed nuts and peanuts
2	Flour, grains, and rice	4	Cereal flour, flakes, grains, and crushed cereal
		5	Rice
		6	Breakfast flakes and muesli
3	Porridge, bread, and pasta	7	Porridge and porridge powder
		8	Soft bread and bread mixes, rye bread and products.
		9	Hard bread, crusts, and flour mixes
		10	Pasta (without filling)
4	Milk, fermented products, and related plant-based products	11	Milk products intended as a drink, and equivalent lactose-free or plant-based variations. (Excluded) *
		12	Fermented milk products not intended for drinking, and equivalent lactose-free or plant-based products
		13	Flavoured fermented milk products not intended for drinking, and equivalent lactose-free or plant-based products
		14	Cream or a mixture of milk and cream, and equivalent lactose-free or plant-based products
		15	Flavoured cream or a mixture of milk and cream, and equivalent lactose-free or plant-based products
5	Cheese and related plant-based products	16	Cheese
		17	Plant-based cheese intended to be used as an alternative to cheese
		18	Fresh cheese and equivalent products.
6	Fats, oils, and spreads	19	Fat spread and blends
		20	Cooking oils, liquid fat spread, and liquid blends
7	Fish, shellfish, and derivative products	21	Fishery products and live mussels
		22	Products of processed fishery products, including sliced cold cuts; smoked or marinated fish; and caviar and other tinned fish products
8	Meat and meat products	23	Unprocessed meat
		24	Products of processed meat, including marinated meat or injection-salted meat; minced meat, sausages, cold-cut sausages, or ground beef; smoked products or cold-cut products
9	Plant-based Products **	25	Products with the same range of uses as fish and meat products above, including sliced sandwich cuts
10	Ready meals	26	Ready meals with vegetables, a protein-containing part, *and* a carbohydrate-containing part
		27	Ready meals with vegetables, a protein-containing part, *or* a carbohydrate-containing part
		28	Pierogies, pizzas, spring rolls, other pies than dessert pies and similar products
		29	Sandwiches, baguettes, wraps, and similar products
		30	Soup (ready-to-eat)
11	Dressings and sauces	31	Dressings (oil- and vinegar-based)
		32	Sauces (ready-to-eat)

#: Group or category number. * Items belonging to group 4, category 11 were excluded from assessment in this study, since the updated Nutri-Score algorithm, published in 2022, did not include drinks and beverages. The group was therefore re-named “fermented products and related plant-based products” for the purposes of this study. ** With the same range of use as meat or fish in groups 7 and 8. This group was re-named “plant-based meat and fish analogues” for the purposes of this study.

## Appendix B. Examples of Differences between the Two Front-of-Pack Nutrition Labels

**Table A2:** Examples of five food items for which a disagreement between the Keyhole and Nutri-Score was found.

Lentil Soup		Keyhole	Nutri-Score 2022		
	Per 100 g	Eligibility (Criterion cut-off)	Points *	Element	*Component*
FVL (%)	41	Yes (≥35%)	1	Favourable	*C* 3 points
Protein (g)	3.8	-	1		
Fibre (g)	3.2	-	1		
Energy (kJ)	263	-	0	Unfavourable	*A* 4 points
Sugar (g)	1.3	-	0		
Sat. fat (g)	0.1	Yes (≤1.5 g/100 g)	0		
Salt (g)	1	No (≤0.8 g/100 g)	4		
Added sugar (g)	0	Yes (≤3 g/100 g)	-	-	-
Grain (%)	0	-	-		
	**Outcome**	**Not Keyhole-eligible**	(Formula *ii*) **Nutri-Score = B**		
**Soy protein kebab, frozen**		**Keyhole**	**Nutri-Score 2022**		
	Per 100 g	Eligibility (Criterion cut-off)	Points *	Element	*Component*
FVL (%)	23	No (≥50%)	0	Favourable	*C* 11 points
Protein (g)	17.4	-	7		
Fibre (g)	7.1	-	4		
Energy (kJ)	136	-	1	Unfavourable	*A* 8 points
Sugar (g)	1.8	Yes (≤3 g)	0		
Sat. fat (g)	0.4	Yes (≤3.5 g)	0		
Salt (g)	1.6	Yes (≤10 g)	7		
Fat (g)	3.8	No (≤1.5 g)	-	-	-
	**Outcome**	**Not Keyhole-eligible**	(Formula *ii*) **Nutri-Score = A**		
**Wholegrain bread, rye** **unsweetened**		**Keyhole**	**Nutri-Score 2022**		
	Per 100 g	Eligibility (Criterion cut-off)	Points *	Element	*Component*
FVL (%)	0	-	0	Favourable	*C* 4 points
Protein (g)	7.1	-	2		
Fibre (g)	5	Yes (≥5 g)	2		
Energy (kJ)	1002	-	2	Unfavourable	*A* 6 points
Sugar (g)	4.3	Yes (≤5 g)	1		
Sat. fat (g)	0.2	-	0		
Salt (g)	0.8	Yes (≤1 g)	3		
Fat (g)	1.55	Yes (≤7 g)	-	-	-
Wholegrain (%)	29	No (≥30%)	-		
	**Outcome**	**Not Keyhole-eligible**	(Formula *ii*) **Nutri-Score = B**		
**Hard cheese, 17% fat**		**Keyhole**	**Nutri-Score 2022**		
	Per 100 g	Eligibility (Criterion cut-off)	Points *	Element	*Component*
FVL (%)	0	-	0	Favourable	*C* 7 points
Protein (g)	30.3	-	7		
Fibre (g)	0	-	0		
Energy (kJ)	1170	-	3	Unfavourable	*A* 18 points
Sugar (g)	0	-	0		
Sat. fat (g)	11.2	-	10		
Salt (g)	1.1	Yes (≤1.6 g)	5		
Fat (g)	17	Yes (≤17 g)	-	-	-
	**Outcome**	**Keyhole-eligible**	(Formula *ii* **^†^**) **Nutri-Score = D**		
**Salmon, cold smoked**		**Keyhole**	**Nutri-Score 2022**		
	Per 100 g	Eligibility (Criterion cut-off)	Points *	Element	*Component*
FVL (%)	0	-	0	Favourable	*C* 7 points
Protein (g)	20	-	7		
Fibre (g)	0	-	0		
Energy (kJ)	724	-	2	Unfavourable	*A* 16 points
Sugar (g)	0	Yes (≤5 g)	0		
Sat. fat (g)	1.4	-	1		
Salt (g)	2.8	Yes (≤3 g)	13		
NFF (%)	6.7	Yes (≤10 g)	-	-	-
Fish composition (%)	97	Yes (≥50%)	-		
	**Outcome**	**Keyhole-eligible**	(Formula *i*) **Nutri-Score = D**		

All items are in the solid food group according to the Nutri-Score 2022 algorithm. For each food item, relevant nutritional parts (e.g., protein, fibre, or salt) are shown per 100 g or as a percentage. The parts are separated into the three “favourable” elements (fruit, vegetable, legume (FVL); protein; fibre) and four “unfavourable” elements (energy; sugar; saturated fat; salt) as per the Nutri-Score 2022 algorithm (see Methods). The parts relevant only to the Keyhole criteria are presented. For Keyhole eligibility, whether each part meets the Keyhole criterion is indicated (yes/no), with the criterion cut-off shown in brackets. For the Nutri-Score, the number of points given to each part is shown, separated by the pre-defined “favourable” and “unfavourable” elements, as per the Nutri-Score 2022 algorithm. The overall points for the *C component* (the sum of points for the “favourable” elements) and overall points for the *A component* (the sum of points for the “unfavourable” elements) is also presented. The outcome (i.e., the Keyhole eligibility and Nutri-Score letter) is presented in the final row. The five items and their respective food groups are: *lentil soup* in the group of ready meals; *soy protein kebab*, *frozen* in the group of plant-based meat and fish analogues; *wholegrain bread*, *rye unsweetened* in the group of porridge, bread, and pasta; *hard cheese*, *17% fat* in the group of cheese and related plant-based products; and *salmon*, *cold smoked* in the group of fish, shellfish, and derived products. FVL: fruit, vegetable (excluding potato), and legume composition; Sat. fat: saturated fat; NFF: non-fish fat (i.e., fat other than fish fat). Formula *i:* Nutri-Score *= A Component − (Points FVL + Points Fibre)* (not including points for protein). Formula *ii:* Nutri-Score *= A Component − C Component* * Point allocation for each part is provided in the Supplementary Material (File S3). **^†^** For cheese items, Formula *ii* is applied.

## References

[1] Kopp W. (2019). How Western Diet and Lifestyle Drive the Pandemic of Obesity and Civilization Diseases. Diabetes Metab. Syndr. Obes..

[2] Greenberg H., Deckelbaum R.J., Eggersdorfer M., Kraemer K., Cordaro J.B., Fanzo J., Gibney M., Kennedy E., Labrique A., Steffen J. (2016). Chapter 2.3: Diet and Non-Communicable Diseases: An Urgent Need for New Paradigms. Good Nutrition: Perspectives for the 21st Century.

[3] WHO (World Health Organization) (2017). Guiding Principles and Framework Manual for Front-of-Pack Labelling for Promoting Healthy Diet.

[4] Franco-Arellano B., Vanderlee L., Ahmed M., Oh A., L’Abbé M. (2020). Influence of Front-of-Pack Labelling and Regulated Nutrition Claims on Consumers’ Perceptions of Product Healthfulness and Purchase Intentions: A Randomized Controlled Trial. Appetite.

[5] (2011). Regulation (EU) No 1169/2011 of the European Parliament and of the Council. https://eur-lex.europa.eu/legal-content/EN/TXT/?uri=CELEX:02011R1169-20180101.

[6] Nohlen H.U., Grammatikaki E., Ciriolo E., Salesse J., Christofoletti M., Bruns J., Marandola F., van Bavel G. (2022). Front-of-Pack Nutrition Labelling Schemes: An Update of the Evidence.

[7] Penzavecchia C., Todisco P., Muzzioli L., Poli A., Marangoni F., Poggiogalle E., Giusti A.M., Lenzi A., Pinto A., Donini L.M. (2022). The Influence of Front-of-Pack Nutritional Labels on Eating and Purchasing Behaviors: A Narrative Review of the Literature. Eat. Weight Disord. Stud. Anorex. Bulim. Obes..

[8] Marandola G., Ciriolo E., van Bavel R., Wollgast J., Storcksdieck genannt Bonsmann (2020). Front-of-Pack Nutrition Labelling Schemes a Comprehensive Review.

[9] European Commission (2020). Farm to Fork Strategy.

[10] Nordic Co-Operation (2022). The Nordic Countries Support the Development of a Harmonised Front-Of-Pack Nutrition Labelling. https://www.norden.org/en/declaration/nordic-countries-support-development-harmonised-front-pack-nutrition-labelling.

[11] Livsmedelverket (The Swedish Food Agency) Tolkningar av Paragrafer Samt Livsmedelsgrupper (Interpretations of Paragraphs and Food Groups). https://kontrollwiki.livsmedelsverket.se/artikel/394/tolkningar-av-paragrafer-samt-livsmedelsgrupper.

[12] Livsmedelverket (The Swedish Food Agency) (2021). FÖreskrifter Om Ändring i Livsmedelsverkets FÖreskrifter (LIVSFS 2005:9) Om Användning Av Viss Symbol.

[13] Wanselius J., Larsson C., Berg C., Öhrvik V., Lindroos A.K., Lissner L. (2022). Consumption of Foods with the Keyhole Front-of-Pack Nutrition Label—Potential Impact on Energy and Nutrient Intakes of Swedish Adolescents. Public Health Nutr..

[14] Livsmedelsverket (The Swedish Food Agency) (2021). Vad Tycker Konsumenterna Om Nyckelhålet?.

[15] Hedengren M., Wassenius M. (2015). A Qualitative Study Concerning the Keyhole’s Influence over 25 Years on Product Development.

[16] van der Bend D.L.M., Lissner L. (2019). Differences and Similarities between Front-of-Pack Nutrition Labels in Europe: A Comparison of Functional and Visual Aspects. Nutrients.

[17] Scientific Committee of the Nutri-Score, Santé Publique France (2022). Nutri-Score Frequently Asked Questions. https://www.santepubliquefrance.fr/media/files/02-determinants-de-sante/nutrition-et-activite-physique/nutri-score/qr-scientifique-technique-en.

[18] Scientific Committee of the Nutri-Score, Santé Publique France (2021). Update of the Nutri-Score Algorithm: Yearly Report from the Scientific Committee of the Nutri-Score. https://sante.gouv.fr/IMG/pdf/annual_report_2021.pdf.

[19] Packer J., Russell S.J., Ridout D., Hope S., Conolly A., Jessop C., Robinson O.J., Stoffel S.T., Viner R.M., Croker H. (2021). Assessing the Effectiveness of Front of Pack Labels: Findings from an Online Randomised-Controlled Experiment in a Representative British Sample. Nutrients.

[20] Pettigrew S., Jongenelis M.I., Jones A., Hercberg S., Julia C. (2023). An 18-Country Analysis of the Effectiveness of Five Front-of-Pack Nutrition Labels. Food Qual. Prefer..

[21] Goiana-Da-Silva F., Cruz-E-Silva D., Nobre-Da-Costa C., Nunes A.M., Fialon M., Egnell M., Galan P., Julia C., Talati Z., Pettigrew S. (2021). Nutri-Score: The Most Efficient Front-of-Pack Nutrition Label to Inform Portuguese Consumers on the Nutritional Quality of Foods and Help Them Identify Healthier Options in Purchasing Situations. Nutrients.

[22] Pauline D., Caroline M., Chantal J., Emmanuelle K.G., Mathilde T., Léopold F., Serge H., Sandrine P. (2015). Effectiveness of Front-of-Pack Nutrition Labels in French Adults: Results from the Nutrinet-Santé Cohort Study. PLoS ONE.

[23] Pettigrew S., Jongenelis M.I., Hercberg S., Julia C. (2022). Front-of-Pack Nutrition Labels: An Equitable Public Health Intervention. Eur. J. Clin. Nutr..

[24] Scientific Committee of the Nutri-Score (2022). Update of the Nutri-Score Algorithm: Update Report from the Scientific Committee of the Nutri-Score. https://sante.gouv.fr/IMG/pdf/maj__rapport_nutri-score_rapport__algorithme_2022_.pdf.

[25] Livsmedelsverket (The Swedish Food Agency) Livsmedelsverkets Livsmedelsdatabas Version 2022-05-24. https://www7.slv.se/SokNaringsinnehall/.

[26] Pointke M., Pawelzik E. (2022). Plant-Based Alternative Products: Are They Healthy Alternatives? Micro- and Macronutrients and Nutritional Scoring. Nutrients.

[27] Mayer Labba I.C., Hoppe M., Gramatkovski E., Hjellström M., Abdollahi M., Undeland I., Hulthén L., Sandberg A.S. (2022). Lower Non-Heme Iron Absorption in Healthy Females from Single Meals with Texturized Fava Bean Protein Compared to Beef and Cod Protein Meals: Two Single-Blinded Randomized Trials. Nutrients.

[28] Bryngelsson S., Moshtaghian H., Bianchi M., Hallström E. (2022). Nutritional Assessment of Plant-Based Meat Analogues on the Swedish Market. Int. J. Food Sci. Nutr..

[29] Naghshi S., Sadeghi O., Willett W.C., Esmaillzadeh A. (2020). Dietary Intake of Total, Animal, and Plant Proteins and Risk of All Cause, Cardiovascular, and Cancer Mortality: Systematic Review and Dose-Response Meta-Analysis of Prospective Cohort Studies. BMJ.

[30] Willett W., Rockström J., Loken B., Springmann M., Lang T., Vermeulen S., Garnett T., Tilman D., DeClerck F., Wood A. (2019). Food in the Anthropocene: The EAT–Lancet Commission on Healthy Diets from Sustainable Food Systems. Lancet.

[31] ProVeg International and the University of Copenhagen (2021). Plant-Based Foods in Europe: How Big is the Market? Smart Protein Plant-Based Food Sector Report by Smart Protein Project, European Union’s Horizon 2020 Research and Innovation Programme (No 862957). https://smartproteinproject.eu/plant-based-food-sector-report/.

[32] Poore J., Nemecek T. (2018). Reducing food’s environmental impacts through producers and consumers. Science.

[33] European Commission Summary of FBDG Recommendations for Starchy Foods for the EU, Iceland, Norway, Switzerland and the United Kingdom. https://knowledge4policy.ec.europa.eu/health-promotion-knowledge-gateway/food-based-dietary-guidelines-europe-table-1_en.

[34] European Commission (2017). Health Promotion and Disease Prevention: Whole Grain.

[35] Mejborn H., Biltoft-Jensen A. (2020). Ernæringsfaglig Vurdering Af Mærkningsordningen Nutri-Score.

[36] Konings J.J.C., Smorenburg H., Roodenburg A.J.C. (2022). Comparison between the Choices Five-Level Criteria and Nutri-Score: Alignment with the Dutch Food-Based Dietary Guidelines. Nutrients.

[37] Söderlund F., Eyles H., Mhurchu C.N. (2020). Stars versus Warnings: Comparison of the Australasian Health Star Rating Nutrition Labelling System with Chilean Warning Labels. Aust. N. Z. J. Public Health.

